# Novel Variants Identified in Multiple Sclerosis Patients From Southern China

**DOI:** 10.3389/fneur.2018.00582

**Published:** 2018-07-25

**Authors:** Hongxuan Wang, Lakhansing Arun Pardeshi, Xiaoming Rong, Enqin Li, Koon Ho Wong, Ying Peng, Ren-He Xu

**Affiliations:** ^1^Department of Neurology, Sun Yat-sen Memorial Hospital,Sun Yat-sen University, Guangzhou, China; ^2^Faculty of Health Sciences, University of Macau, Taipa, Macau

**Keywords:** multiple sclerosis, whole-exome sequencing, single nucleotide polymorphism, human leucocyte antigen, TRIOBP

## Abstract

**Background:** Multiple sclerosis (MS) is an autoimmune and demyelinating disease. Genome-wide association studies have shown that MS is associated with many genetic variants in some human leucocyte antigen genes and other immune-related genes, however, those studies were mostly specific to Caucasian populations. We attempt to address whether the same associations are also true for Asian populations by conducting whole-exome sequencing on MS patients from southern China.

**Methods:** Genomic DNA was extracted from the peripheral blood mononucleocytes of 8 MS patients and 26 healthy controls and followed by exome sequencing.

**Results:** In total, 41,227 variants were found to have moderate to high impact on their protein products. After filtering per allele frequencies according to known database, 17 variants with the allele frequency <1% or variants with undetermined frequency were identified to be unreported and have significantly different frequencies between the MS patients and healthy controls. After validation via Sanger sequencing, one rare variant located in exon 7 of *TRIOBP* (Chr22: 37723520G>T, Ala322Ser, rs201693690) was found to be a novel missense variant.

**Conclusion:** MS in southern China may have association with unique genetic variants, our data suggest *TRIOBP* as a potential novel risk gene.

## Introduction

Multiple sclerosis (MS) is one of the chronic inflammatory demyelinating diseases of the central nervous system (1). Although the causes of MS are still largely unclear, it is thought to be the consequence of the interaction of genetic and environmental factors (1–3). Epidemiological studies have shown that MS is much less prevalent in East Asia than Europe and North America (4–7), which may be attributed to different genetic bases underlying the susceptibility to MS in various populations.

For the past several decades, many genetic variants have been found to be associated with MS in the western populations (8–10). However, lots of risk variants could not be validated in Asian populations (2, 11). Furthermore, not only common variants but rare variants in protein-coding regions also play critical roles in the development of complex diseases (12, 13). In this study, we determined the genetic differences between MS patients and healthy controls in southern China using whole-exome sequencing (WES) to find MS-associated variants in this area.

## Materials and methods

### Patients and samples

The study was reviewed and approved by both ethic committees of Sun Yat-sen Memorial Hospital of Sun Yat-sen University and University of Macau. Patients with MS were diagnosed according to 2010 revision of the McDonald criteria (14) and were recruited from Jan. 1, 2016 to Dec. 31, 2016 in Sun Yat-sen Memorial Hospital. Healthy volunteers who were age- and gender-matched to the MS patients and had no history of autoimmune disorders, tumors, and other chronic illness such as hypertension and diabetes were also recruited. All MS patients and healthy volunteers were southern Chinese and included into the subsequent analysis after they had completed a written informed consent. Patients with underdetermined diagnoses, previous history of transplantation, previous plasmapheresis or stem cell therapy were excluded from the study. Peripheral whole blood was collected for preparing DNA libraries. Genomic DNA was extracted from peripheral blood mononucleocytes of each individual patient or healthy donor with a DNA extraction kit (Tiangen Biotech). Then, genomic DNA was sonicated into fragments of around 200 bp for preparing DNA libraries. DNA libraries for Illumina sequencing were constructed using the NEB Ultra II DNA Prep Kit (NEB) followed by quality verification via DNA High-Sensitivity Bioanalyzer assay (Agilent).

### Whole-exome sequencing and confirmation via sanger sequencing

Exome capture was performed with the TruSeq Rapid Exome Prep kit (Illumina). Multiple individual DNA libraries with different Illumina sequencing indexes were pooled together for exome capture and subsequent sequencing. Exon-enriched DNA library pools were sequenced by Illumina HiSeq 2500 platform (15). FastQC (16) tool was used to check the quality of raw fastq data generated by sequencing. The raw sequencing reads were aligned to the Ensembl (release 84) Human Genome Build 38 (GRCh38.p5) (17) using the Burrows-Wheeler Alignment tool (18). Reads which mapped to multiple locations were marked as duplicate using Picard tool (BroadInstitute)^1^ for future filtering during variant calling. Local realignment of reads around indels and base quality score recalibration was performed using the IndelRealigner and BaseRecalibrator modules of the Genome Analysis Toolkit (GATK) (19, 20). Finally, variants were called using HaplotypeCaller module and variant quality scores were recalibrated with VariantRecalibrator module of GATK.

True variants were identified only when the sequencing read depth of the mutation was equal to or more than 10 reads. SnpEff (21) was used for the general variant annotation purpose, and SnpSift (22) was used to add additional annotation from the 1000 Genomes Project (1KGP) (23), Exome Aggregation Consortium (ExAC) (24), and dbNSFP (25) variant databases. The variants which were predicted by SnpEff to have moderate or high impact on the gene products were screened for further analysis. Allele frequencies from both the 1KGP and ExAC were used as reference allele frequency in the different populations. For TRIOBP, the identified variants were confirmed through Sanger sequencing using target-specific primers as follows: forward GGACAGCACTGGGCAAGG, and reverse GGGAGTACAAGTAGGAAAAGAA.

### Statistical analysis

Variant frequencies were described as proportions, and comparisons of frequencies between different groups were analyzed using Fisher's exact tests. Two-tailed *P* < 0.05 was used as statistically significance.

## Results

### Characteristics of samples

Eight MS patients and 26 healthy controls were included in the study. All the eight patients met the McDonald criteria (2010 revision) for MS, six of them were diagnosed as relapsing-remitting MS (RRMS) who had more than one clinical attack and had one or more typical lesions in two or more typical MS-affecting areas of the central nervous system (CNS), and the other two patients diagnosed as clinically isolated syndrome with more than one objective lesion in two or more typical MS-affecting areas and oligoclonal bands in cerebral spinal fluid. All the patients and healthy volunteers came from several provinces in southern China including Guangdong, Hunan, Guangxi and Fujian, which are within 1,000-km away from the hospital. All the healthy controls had no relative relationship with the patients. The MS patients were 31.63 ± 15.45 years old on average, while the healthy controls 31.24 ± 8.52 years old (*P* > 0.05). Female-to-male ratio happened to be 1:1 for the recruited MS patients (which is lower than the ratio for MS patients in general with *P* > 0.05 per Fisher's Exact Test) and 15:11 for the healthy controls.

### Rare variants revealed via whole-exome sequencing

On average, 2 × 37,614,484 pair-end reads (100 bp of read length) were generated after raw sequencing data filtering, mapping, and realignments, and per-base coverage of target exome regions was around 167 × per person. About 87.4% of exomes had sequencing depth > 1×, and 58.2% of exomes had sequencing depth > 10×.

**Figure 1:**
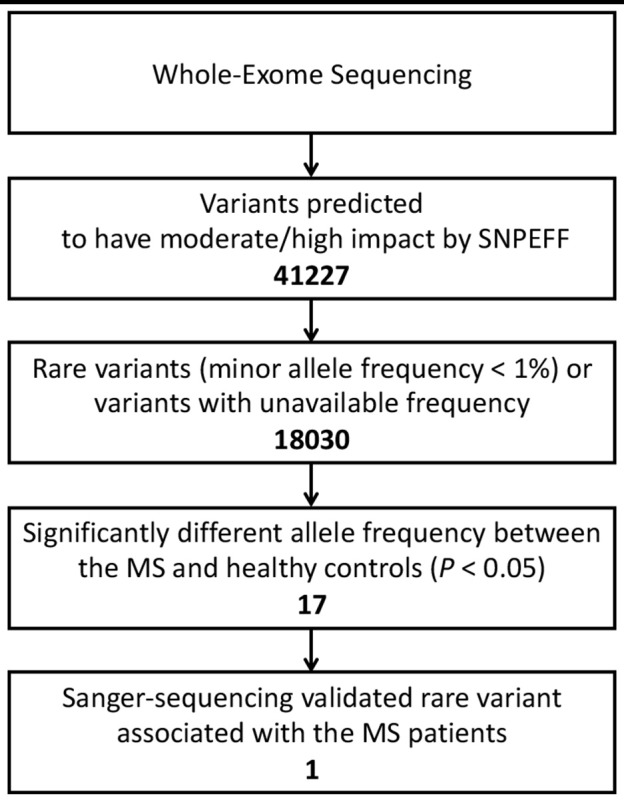
Workflow for data processing, and variant calling and filtering.

**Figure 2:**
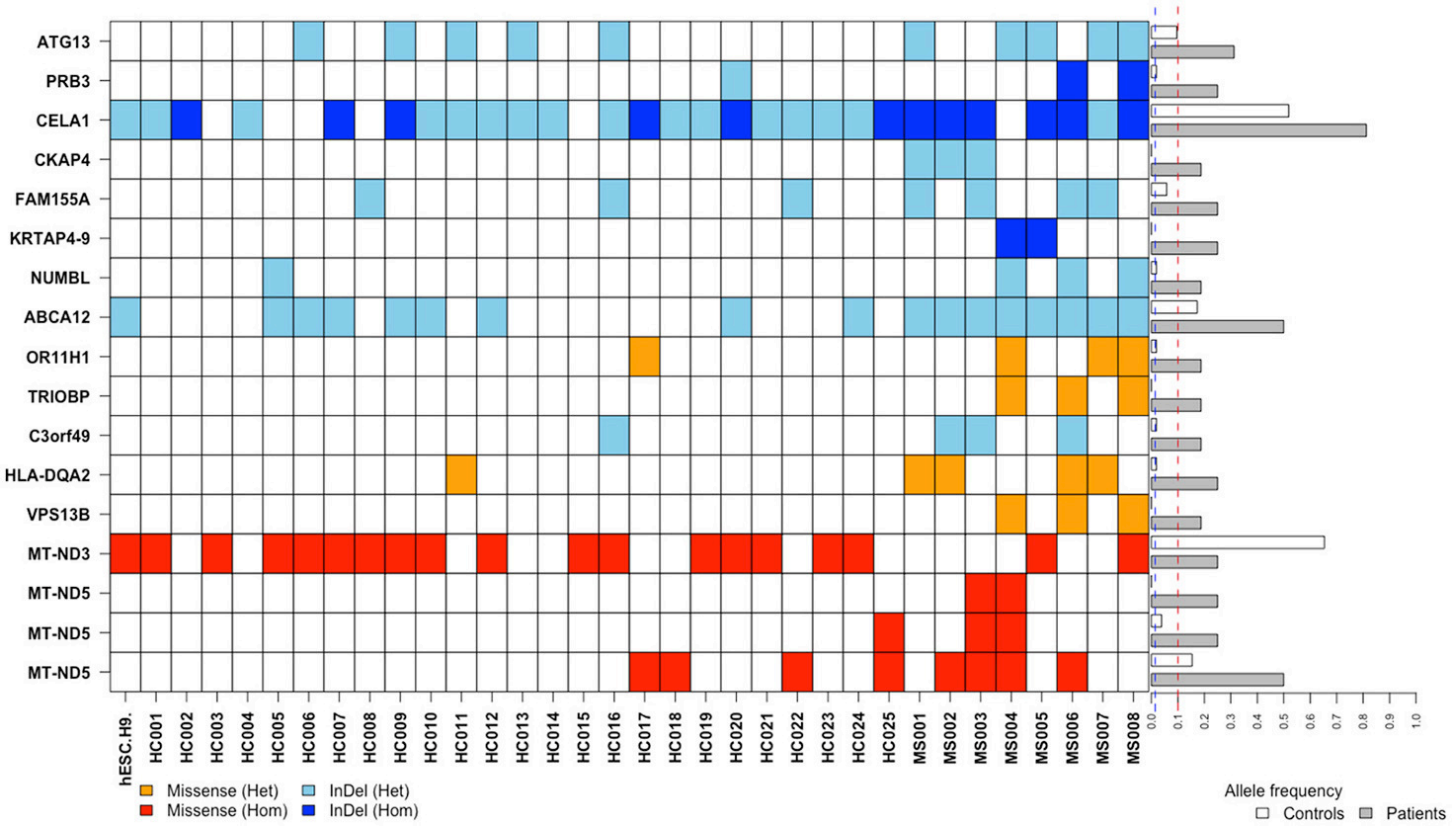
Distributions and frequencies of 17 rare and frequency-unavailable variants associated with the MS patients. Blocks in various colors indicate specific allele types (designated below) in specific loci of corresponding genes (listed in the left side) in each healthy control (HC) or MS patient (MS) (listed at the bottom). The horizontal bars in the right side indicate minor allele frequencies in the healthy controls (white bars) and MS patients (gray bars), respectively. Across these bars are two vertical and dashed lines in blue and red representing allele frequency of 1 and 10%, respectively.

After annotations for all variants according to SnpEff database, a total of 41,227 variants which may have moderate or high impact on their translation products were found in the MS patients and healthy controls. We then filtered out common variants and identified 18,030 rare variants (with allele frequency <1% referring to both 1KGP/ExAC and East Asian populations of 1KGP/ExAC) or variants with undetermined allele frequency. Among the 18,030 variants, 17 variants (located in 15 genes) were identified with allele frequencies significantly different between the MS patients and healthy controls (*P* < 0.05) (Table 1, Figures 1, 2).

**Table 1:** Rare or frequency-unavailable variants associated with MS patients.

**Position**	**SNP ID**	**Allele change**	**Gene**	**Variant type**	**Depth**	**Het/Hom in HC (n)**	**AF_HC (%)**	**Het/Hom in MS (n)**	**AF_MS (%)**	***P*-value**	**Reference MAF (%)^a^**
Chr11:46644265		CT>C,CTT	ATG13	Splice acceptor	1,254	5/0	9.62	5/0	31.25	0.0474	FU
Chr12:11267400		A>AG	PRB3	Frameshift	1,064	1/0	1.92	0/2	25	0.0095	FU
Chr12:51346631		A>AAG	CELA1	Frameshift	2,899	15/6	51.92	1/6	81.25	0.0454	FU
Chr12:106247620		TGCC>T	CKAP4	Inframe deletion	671	0/0	0.00	3/0	18.75	0.0112	FU
Chr13:107866338	rs537066337	TCTG>TCTGCTG,T,GCTG	FAM155A	Inframe insertion	950	3/0	5.77	4/0	25	0.0479	0.359^b^
Chr17:41105490		CTG…TGT>C	KRTAP4-9	Splice acceptor	1,325	0/0	0.00	0/2	25	0.0022	0.544
Chr19:40667999		TTGC>T	NUMBL	Inframe deletion	1,572	1/0	1.92	3/0	18.75	0.0380	FU
Chr2:215075560		GA>G	ABCA12	Frameshift	1,527	9/0	17.31	8/0	50	0.0176	FU
Chr22:15528427	rs202150076	C>T	OR11H1	Missense	2,847	1/0	1.92	3/0	18.75	0.0380	0.469^c^
Chr22:37723520	rs201693690	G>T	TRIOBP	Missense	2,291	0/0	0.00	3/0	18.75	0.0112	0.047
Chr3:63831135	rs3830344	C>CACACT	C3orf49	Frameshift	1,958	1/0	1.92	3/0	18.75	0.0380	0.246
Chr6:32745313	rs752313403	T>A	HLA-DQA2	Missense	3,123	1/0	1.92	4/0	25	0.0095	0.044
Chr8:99854174		A>C	VPS13B	Missense	1,322	0/0	0.00	3/0	18.75	0.0112	FU
ChrMT:10398		A>G	MT-ND3	Missense	5,651	0/17	65.38	0/2	25	0.0083	FU
ChrMT:12338		T>C	MT-ND5	Missense	6,279	0/0	0.00	0/2	25	0.0022	FU
ChrMT:13708		G>A	MT-ND5	Missense	7,017	0/1	3.85	0/2	25	0.0242	FU
ChrMT:13928		G>C	MT-ND5	Missense	7,672	0/4	15.38	0/4	50	0.0078	FU

SNP, single nucleotide polymorphism; Het, heterogenic allele change; Hom, homogenic allele change; AF, allele frequency; MAF, minor allele. HC, healthy control; MS, MS patients; FU, frequency unavailable.

a Reference allele frequency from ExAC;

b Reference allele frequency from 1KGP;

c *Reference allele frequency from East Asian of ExAC*.

### A MS-associated missense in *TRIOBP*

**Figure 3:**
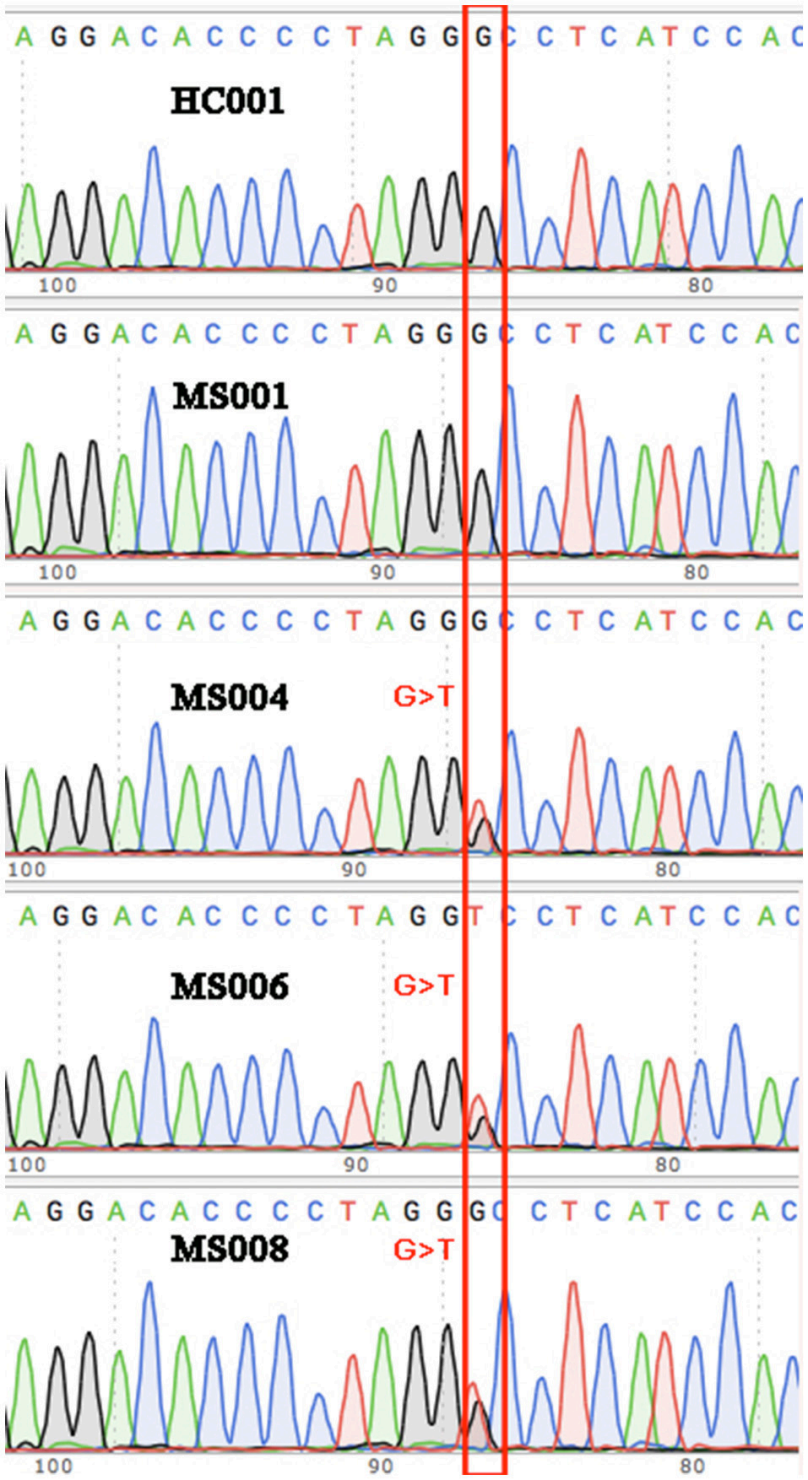
Validation via Sanger sequencing. The G>T variant identified in three of the eight MS patients was verified via Sanger sequencing. Wild-type sequences were shown for some of the other MS patients and the healthy controls as representatives.

Among the 17 variants (possibly associated with MS) identified in our study, 6 were known rare variants. We validated the variants by Sanger sequencing (Figure 3), and found that one of the variants in the gene *TRIOBP* had significantly different allele frequency between the MS patients and healthy controls (*P* < 0.05).

The variant is a missense (Chr22: 37723520G>T, Ala322Ser, rs201693690) located in the exon 7 of *TRIOBP*, causing an amino acid substitution (Ala322Ser). Among the eight MS patients, three had heterogeneous variants in Chr22: 37723520 (G/T). It has much higher allele frequency in the MS patients than the healthy controls (18.75 vs. 0%, *P* = 0.0112). This variant has been documented in the ExAC study, and found to be associated with deafness and schizophrenia. The frequency of allele T in Chr22:37723520 is only 0.047% in the total population of the ExAC database, indicating that it is a rare variant in human genome (Figure 2).

## Discussion

In this study, we recruited 8 MS patients and 26 healthy controls in southern China. Due to the low morbidity of MS in southern China, we could not recruit more patients within the two-year study period. Following WES, we found 17 rare variants or variants with unknown allele frequency had significantly different allele frequencies between the MS patients and healthy controls, including a missense in *TRIOBP* (Chr22: 37723520G>T, Ala322Ser, rs201693690). *TRIOBP* may be a novel risk gene among southern Chinese.

The association between gene polymorphisms and MS has been investigated for decades. The most studied polymorphisms with MS are the human leukocyte antigen (HLA) genes. It has been found that MS risk is strongly associated with HLA-DRB1^*^15:01 and the related haplotype HLA-DRB1^*^15:01-DQA1^*^01:02-HLA-DQB1^*^06:02 in Caucasian populations such as Europeans and North Americans (2, 26). However, it is difficult to associate other HLA-related risk alleles with MS because strong linkage disequilibrium is present in the HLA-DRB1-DQA1-DQB1 regions (2). It is also difficult to confirm risk alleles in genes unrelated to HLA since their genetic effects may be much smaller than that of HLA-DRB1^*^15:01 (2). Furthermore, the associations of HLA-DR allele polymorphisms with MS found in Caucasian populations do not consistently apply to the Asian populations including Chinese. For example, HLA-DRB1^*^15:01 is not strongly associated with MS in Asian populations (11). The association of HLA-DRB1^*^15:01 with MS risk was weaker among northern Chinese than Caucasians, whereas no association of HLA-DRB1^*^15:01 with MS risk was found in southern Chinese (27). These findings indicate that polymorphisms in HLA-unrelated genes might be associated with MS among southern Chinese.

Recently, several genome-wide association studies (GWAS) with large numbers of samples had identified 57 non-HLA SNPs to be associated with MS (10). However, the populations involved in the study were mainly Caucasian populations. Another GWAS study from an Australian group showed that genetic loci for Epstein-Barr virus nuclear antigen-1 were positively associated with the risk of MS (28). However, the association cannot be found in Asian populations (29). These results suggest that MS-related genetic heterogeneity exists in different ethnic populations, and new studies on Asian populations are needed to identify genetic bases related to the development of MS in Asia. We performed the exome-sequencing study to discover whether any MS-associated variants differ between southern Chinese and Caucasians.

WES is one of the powerful next-generation sequencing techniques to explore global genetic variants in Mendelian inheritance disorders and many other complex diseases (30–32). It allows discovery of rare variants in coding sequences that may cause mutations in their protein products and subsequent disease phenotypes (31, 32) or contribute to heritability of complex traits. In this study, WES has revealed that the frequency of the rare variant in *TRIOBP* is significantly different between the MS patients and healthy controls, and its allele frequency is much higher than that of reference in known databases (18.75 vs. 0.047% in ExAC). And subsequent Sanger sequencing validated the variant in our samples. Although our sample size is very small, the much higher allele frequency in the MS patients than the healthy controls suggests that this variant may be associated with the risk of MS development in southern China.

This variant, which has not been identified in any MS GWAS studies, may play an important role in the development of MS among southern Chinese. *TRIOBP* is a gene encoding TRIO and F-actin binding protein, and locates in chromosome 22. The encoded protein interacts with trio, which regulates actin cytoskeleton organization, cell migration and cell growth, and it also stabilizes F-actin structures (33, 34). Previous studies have found that mutations or variants of *TRIOBP* cause genetic sensorineural hearing impairments (35, 36), and abnormal TRIOBP protein aggregation leads to chronic mental illness (37, 38). However, the mechanism for how TRIOBP affects immune or inflammation is unknown.

Our exome-sequencing also identified several variants in the other genes which have higher allele frequencies in the MS patients than the normal controls. However, there is no referable information for the allele frequency for some of the variants (e.g., *CKAP4, NUMBL*, and *VPS13B*). The other variants such as the missense in *HLA-DQA2* (Chr6: 32745313T>A, rs752313403), which had much higher allele frequency in the MS patients than the healthy controls, could not be validated through Sanger sequencing. Thus, we cannot include it as a risk variant.

Furthermore, other etiologic factors can trigger MS or increase the susceptibility to MS. For example, it has been proposed that *HLA2TA* mRNA level can be reduced by the active replication of human herpesvirus 6 in MS patients (39, 40). It is intriguing to determine whether these external etiologic factors are differentially distributed among various populations. Simultaneous testing of both genetic and environmental etiologic factors may further elucidate the correlations.

In conclusion, this study on MS patients from southern China has identified a missense rare variant in *TRIOBP* (Chr22: 37723520G>T, Ala322Ser, rs201693690) that may be associated with MS. Further study is necessary to verify the above findings in a larger sample size, and animal models are needed to confirm the role of this and other potential variants.

## Author contributions

HW contributes to recruit study participants, collect samples, prepare exome sequencing samples, and write manuscript. LP contributes to analyze exome sequencing samples. XR contributes to recruit study participants and collect samples. EL contributes to prepare exome sequencing samples. KW contributes to prepare exome sequencing samples and analyze exome sequencing samples. YP contributes to supervise whole project, design the study, recruit study participants, collect samples, and write manuscript. R-HX contributes to supervise whole project, design the study, prepare exome sequencing samples, and write manuscript.

### Conflict of interest statement

The authors declare that the research was conducted in the absence of any commercial or financial relationships that could be construed as a potential conflict of interest.

## Footnotes

^1^Broadinstitute Picard Tools - By Broad Institute. http://broadinstitute.github.io/picard/index.html
